# Effect of an E-Learning Module on Personal Protective Equipment Proficiency Among Prehospital Personnel: Web-Based Randomized Controlled Trial

**DOI:** 10.2196/21265

**Published:** 2020-08-21

**Authors:** Laurent Suppan, Mohamed Abbas, Loric Stuby, Philippe Cottet, Robert Larribau, Eric Golay, Anne Iten, Stephan Harbarth, Birgit Gartner, Mélanie Suppan

**Affiliations:** 1 Division of Emergency Medicine Department of Anesthesiology, Clinical Pharmacology, Intensive Care and Emergency Medicine University of Geneva Hospitals and Faculty of Medicine Geneva Switzerland; 2 Infection Control Program and WHO Collaborating Centre on Patient Safety University of Geneva Hospitals and Faculty of Medicine Geneva Switzerland; 3 Genève TEAM Ambulances Geneva Switzerland; 4 Division of Anesthesiology Department of Anesthesiology, Clinical Pharmacology, Intensive Care and Emergency Medicine University of Geneva Hospitals and Faculty of Medicine Geneva Switzerland

**Keywords:** personal protective equipment, COVID-19, electronic learning, prehospital, randomized controlled trial, protection, equipment, safety, gamified, online learning, communication

## Abstract

**Background:**

To avoid misuse of personal protective equipment (PPE), ensure health care workers’ safety, and avoid shortages, effective communication of up-to-date infection control guidelines is essential. As prehospital teams are particularly at risk of contamination given their challenging work environment, a specific gamified electronic learning (e-learning) module targeting this audience might provide significant advantages as it requires neither the presence of learners nor the repetitive use of equipment for demonstration.

**Objective:**

The aim of this study was to evaluate whether a gamified e-learning module could improve the rate of adequate PPE choice by prehospital personnel in the context of the coronavirus disease (COVID-19) pandemic.

**Methods:**

This was an individual-level randomized, controlled, quadruple-blind (investigators, participants, outcome assessors, and data analysts) closed web-based trial. All emergency prehospital personnel working in Geneva, Switzerland, were eligible for inclusion, and were invited to participate by email in April 2020. Participants were informed that the study aim was to assess their knowledge regarding PPE, and that they would be presented with both the guidelines and the e-learning module, though they were unaware that there were two different study paths. All participants first answered a preintervention quiz designed to establish their profile and baseline knowledge. The control group then accessed the guidelines before answering a second set of questions, and were then granted access to the e-learning module. The e-learning group was shown the e-learning module right after the guidelines and before answering the second set of questions.

**Results:**

Of the 291 randomized participants, 176 (60.5%) completed the trial. There was no significant difference in baseline knowledge between groups. Though the baseline proportion of adequate PPE choice was high (75%, IQR 50%-75%), participants’ description of the donning sequence was in most cases incorrect. After either intervention, adequate choice of PPE increased significantly in both groups (*P*<.001). Though the median of the difference in the proportion of correct answers was slightly higher in the e-learning group (17%, IQR 8%-33% versus 8%, IQR 8%-33%), the difference was not statistically significant (*P*=.27). Confidence in the ability to use PPE was maintained in the e-learning group (*P*=.27) but significantly decreased in the control group (*P*=.04).

**Conclusions:**

Among prehospital personnel with an already relatively high knowledge of and experience with PPE use, both web-based study paths increased the rate of adequate choice of PPE. There was no major added value of the gamified e-learning module apart from preserving participants' confidence in their ability to correctly use PPE.

## Introduction

### Background and Importance

Adequate use of personal protective equipment (PPE) is of paramount importance to ensure health care workers’ safety and to avoid shortages of such equipment in the context of the coronavirus disease (COVID-19) pandemic [1,2]. Protection guidelines against severe acute respiratory syndrome coronavirus 2 (SARS-CoV-2) infection have rapidly evolved following publication of new evidence regarding its mode of transmission, making prompt adaptation of the guidelines both frequent and necessary [3-5]. Prehospital personnel is particularly at risk of contamination as they usually work in a challenging environment and have to stay next to their patients in the narrow space of the ambulance for extended periods of time. This risk is further increased when high-risk procedures such as endotracheal intubation have to be performed [6-8].

To avoid misuse of PPE, effective communication of the corresponding guidelines to frontline health care workers is necessary [9]. However, continuous education has been massively disrupted due to the cancellation of continuous education sessions to limit disease transmission [10]. In this challenging context, electronic learning (e-learning) might provide significant advantages as it requires neither the presence of learners nor the repetitive use of equipment for demonstration as could be the case for live simulation [11,12].

### Goal of This Investigation

The purpose of this study was to evaluate whether a specifically designed gamified e-learning module [13] could improve the rate of adequate PPE choice by prehospital personnel in the context of the COVID-19 pandemic. Our hypothesis was that knowledge of PPE guidelines would be inconsistent between prehospital personnel, and that an e-learning module may increase and standardize knowledge regarding the use of PPE. This could help limit both underuse and overuse of such equipment.

## Methods

### Study Design

**Figure 1:**
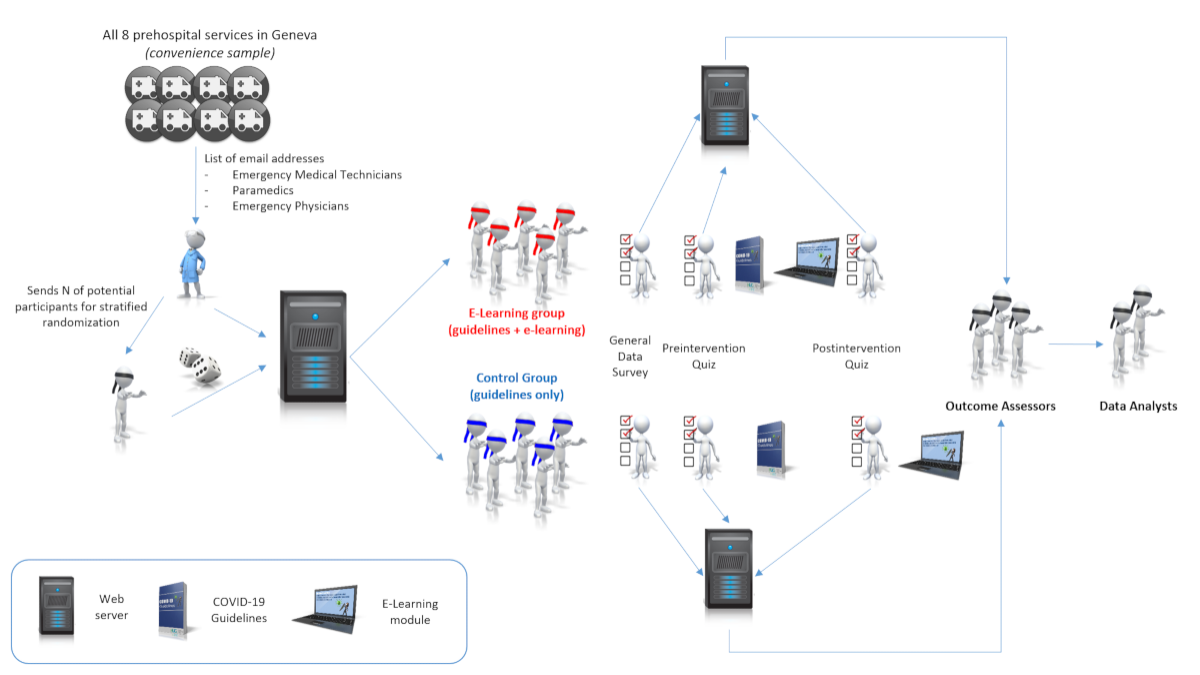
Study design.

This was an individual-level, stratified, randomized, controlled, quadruple-blind (investigators, participants, outcome assessors, and data analysts) closed web-based trial (Figure 1) designed following the CONSORT-EHEALTH (Consolidated Standards of Reporting Trials of Electronic and Mobile HEalth Applications and onLine TeleHealth) guidelines [14,15] and incorporating relevant elements from the CHERRIES (Checklist for Reporting Results of Internet E-Surveys) statement [16].

The regional ethics committee issued a “Declaration of no objection” (Req-2020-00374) as the population studied was not considered vulnerable according to the Swiss federal law on human research [17]. As the purpose of the study was to examine the effect of two different study paths only on providers’ knowledge and attitude toward PPE, registration of the trial was not performed as it is not deemed necessary by the International Committee of Medical Journal Editors [18].

### Setting

The study took place in April 2020 in Geneva, Switzerland; the detailed organization of local prehospital emergency services has already been described elsewhere [19]. There are five levels of increasing expertise working in the prehospital field. Ambulance drivers all have a basic life support certificate but are never called upon to deal with emergency situations. Emergency medical technicians (EMTs) are certified after 1 year of training, and can either transport stable patients on their own, or team up with a paramedic to deal with more difficult prehospital situations. The highest level of nonphysician care is provided by paramedics, who complete a 3-year curriculum. Whenever a potentially life-threatening emergency is identified by emergency dispatchers, paramedics are sent in teams of two. A medical reinforcement by way of a light vehicle, Service Mobile d’Urgence et de Réanimation (SMUR), staffed by a paramedic and an emergency physician can either be dispatched at the same time as the ambulance or be called upon by paramedics if specialized medical care is required on site. Emergency physicians are either senior residents or fellows working in one of the following departments: emergency medicine, anesthesiology, or general internal medicine. They can be supervised, either by call or on-site, by a senior specialist emergency physician, which represents the highest level of prehospital care in this setting.

Apart from the physician-staffed prehospital medical service, there are seven different ambulance companies in Geneva, two of which belong to public organizations while the remaining five are privately owned. As each company has its own continuous education structure as well as its own equipment, medical devices, and protocols, one cannot expect all paramedics to share the same knowledge level regarding all aspects of prehospital emergencies.

### Online Platform

An online platform [20] developed under the Joomla! 3.9 content management system (Open Source Matters) was created specifically for the purpose of this study. A mailing management component (AcyMailing 5.10, Acyba), a survey component (Community Surveys Pro 5.4.0, CoreJoomla), and a form builder component used to issue completion certificates (BreezingForms Pro 1.9.0, Crosstec) were installed on the website. L Suppan was the only author with access to the platform’s administration console. No maintenance or update was planned on the server, platform, or content during the study period.

### Study Material

A previously described gamified e-learning module created under Storyline 3 (Articulate Global) was used in this study [13]. The module contains 19 sections and embeds 7 video sequences. Within the module, trigger mechanisms are used to check that the user had accessed and completed all required steps before being allowed to proceed to the following section. The e-learning package is available on the study website where it can be viewed and downloaded freely. The comprehensive prehospital COVID-19 guidelines from the Geneva University Hospitals, version 1.11, was also used in this study [21].

Two quizzes were created by BG and L Suppan: a preintervention quiz designed to establish the participants’ baseline knowledge regarding PPE, their use and indication, and a postintervention quiz to assess whether these parameters had changed. Both quizzes contained 10 closed questions, either multiple choice or multiple answer. Questions designed to assess PPE choice were preceded by short clinical scenarios. Each quiz was displayed over 5 pages. The number of questions was limited to reduce attrition. These quizzes were tested and validated by all coinvestigators.

Consistency of specific “free-text” questions, such as age, was checked by means of regular expression (“regex”) rules. All answers were automatically checked for completeness by the system before participants were allowed to proceed to the next page. Custom validation messages were displayed to inform users who had not answered a question. Participants were not allowed to correct or review their answers once a page had been completed.

### Subjects and Inclusion and Exclusion Criteria

Chief ambulance officers of all services were asked to provide one of the investigators (L Suppan) with a list of all the professional email addresses of their EMTs, paramedics, and emergency physicians. All the email addresses received from these officers were included.

Email addresses of ambulance drivers were excluded as these drivers usually only deal with interhospital transfers and almost never don PPE. Senior specialist emergency physicians were also excluded, as they are few in number and are usually involved in the writing of the guidelines or in the creation of the learning material; in addition, some of them are authors of this study. Finally, the email addresses of the paramedics who participated in the creation of this study or the learning material were also excluded.

### Randomization and Allocation Concealment

Before performing a 1:1 randomization, stratification was achieved according to professional status (EMTs, paramedics, and emergency physicians). Email addresses were then sorted according to alphabetical order, and an investigator (MS) who did not have access to the email addresses database was given the number of participants by category and performed the randomization using a computer-generated table. The randomization key was then combined with the list of email addresses and entered in the mailing component by the only investigator who had access to the system (L Suppan). As the list of email addresses and allocations were solely present in an encrypted database, all other investigators were blinded as to group allocation.

### Enrolment and Consent

Individual emails that were identical for all participants except for the unique links that pointed to one of the two study paths were sent on April 13, 2020 (Multimedia Appendices 1 and 2). These unique links were automatically created by the survey component but were not recorded in the mailing component other than in generic form (there was no way the individual tokens could be linked to the email addresses). The webmaster could therefore only know whether the email had been opened and the survey started, but was prevented from reconciling the email address with the survey answers, thereby guaranteeing user anonymization. Using unique links served two purposes: they allowed the participants to resume the course in case they were interrupted and also avoided double entries. Given the current circumstances, and as the vast majority of prehospital professionals had had their holidays cancelled and were therefore at work, participants were only given 12 days to complete the study, with email reminders sent on days 3, 7, and 10.

Apart from the survey link, the emails contained information regarding the study length and objectives as well as a short data protection statement. Participants were informed that they would be presented with the most recent version of the prehospital COVID-19 guidelines as well as with an e-learning module, though the order in which these materials would be shown was not explained. They were informed that, by clicking on the survey link, they consented to participate in the study and were provided with the names and electronic addresses of five investigators (BG, EG, L Stuby, L Suppan, and PC), whom they could contact at any time. As collected data were irreversibly anonymized, it was impossible for users to ask for their own answers to be deleted once the survey had been completed.

To improve the response rate, the chief ambulance and medical officers of all companies were asked to encourage their paramedics, EMTs, and emergency physicians to participate in the study. Participation reminders were also sent to all prehospital personnel along with a daily COVID-19 information newsletter.

Participation was not mandatory. No monetary incentive or prize was offered to the survey participants. As the e-learning module was akin to a continuous education session, participants were informed before beginning the survey that they would be able to print a continuous education certificate upon completion. As the certificate component was independent from both the mailing and the survey components, participants were ensured they could generate the certificate without their identity being disclosed.

### Study Sequence

After clicking on the survey link, a welcome screen containing detailed information about the study was displayed. This welcome screen was identical for both groups and, similarly to the email messages, did not convey any information regarding the study sequence to ensure the participants were adequately blinded.

After clicking on the start button, participants were asked a series of questions designed to gather demographic-related data. Adaptive questioning was used in this section to avoid displaying irrelevant questions. Participants were then asked a series of general questions related to SARS-CoV-2 and the COVID-19 pandemic.

The control group was then shown the prehospital COVID-19 guidelines. They were then asked to answer a second set of questions before being prompted to evaluate the learning path up to this point. Only then could they access the e-learning module and download their certificate.

The e-learning group followed the same path at first, but accessed the e-learning module immediately after being shown the guidelines. This group then completed the same second set of questions, was asked to evaluate the learning path (which in this case included the e-learning module), and was finally allowed to download the completion certificate.

### Outcomes

The main outcome was defined as the difference in the proportion of correct choice of PPE before and after the course, assessed by means of short clinical scenarios.

Secondary outcomes were stratification of the main outcome by profession and by personal history of COVID-19 (whether or not the participant had been infected), accuracy of donning and doffing sequences reconstitutions, differences in the rates of overuse and underuse of PPE, confidence in one’s ability to use PPE, perceived usefulness of the course, and satisfaction regarding the course. The latter three outcomes were assessed by means of a 5-point Likert scale.

### Data Collection

Data was electronically recorded and securely stored in an encrypted MariaDB database (Version 5.5.5; MariaDB Foundation). At the end of the study, all data was extracted to CSV format by the only investigator who had access (L Suppan). No personally identifiable data (including name, date of birth, email, or IP address) was ever asked for or recorded.

### Data Curation and Availability

The extracted data were imported into Stata (StataCorp LLC). Variables were renamed to facilitate their understanding by the blinded data analysts. All data that could have enabled data analysts to identify group allocation were removed, and data fields were sorted accordingly. Incomplete questionnaires were excluded at this stage. The control and e-learning groups were renamed using city names (Moscow and Nairobi), and all relevant data were exported by L Suppan to a Stata .dta file and sent to L Stuby and MA for analysis.

### Sample Size

As guidelines differ from region to region, we decided to only include prehospital staff working in the Geneva emergency medical system, thereby using a convenience sample rather than performing a sample size calculation.

### Outcomes Assessment

Though most outcomes were electronically recorded and their interpretation therefore was generally independent of subjective human evaluation, comments had to be assessed by outcome assessors. Two assessors (L Stuby and PC), blinded as to group allocation, were asked to independently assess all comments. The nature of comments were to be rated as “positive,” “negative,” or “neutral” regarding the study, and as to whether they challenged Infection Prevention and Control (IPC) guidelines (binary, yes versus no). Disagreements were solved by sending the unclear comments to a third outcome assessor (BG), who was blinded to the previous assessments.

### Statistical Analysis

Statistical analysis was performed using Stata 15. Continuous independent outcomes were assessed using the Student *t* test or the Mann-Whitney rank-sum test depending on normality. Categorical outcomes were assessed using Fisher exact test. A two-sided *P*<.05 was considered significant. Normality of distribution was first assessed graphically. In case of doubt, the Shapiro-Wilk test was applied.

Continuous paired data were assessed using either the paired Student *t* test or the Wilcoxon matched-pairs signed-rank test depending on normality. The sign test for matched pair was used if symmetry could not be proven. Categorical paired data were analyzed using asymptotic symmetry and marginal homogeneity tests. Binomial paired data were assessed using the McNemar test.

Stratification was defined a priori based on expertise level (EMTs, paramedics, or physicians) and COVID-19 status (negative; confirmed, quarantined; confirmed, cured or unknown). Two post hoc sensitivity analyses were conducted.

### Data Availability

The original data has been deposited to Mendeley Data [22].

## Results

### Characteristics of Study Subjects

**Figure 2:**
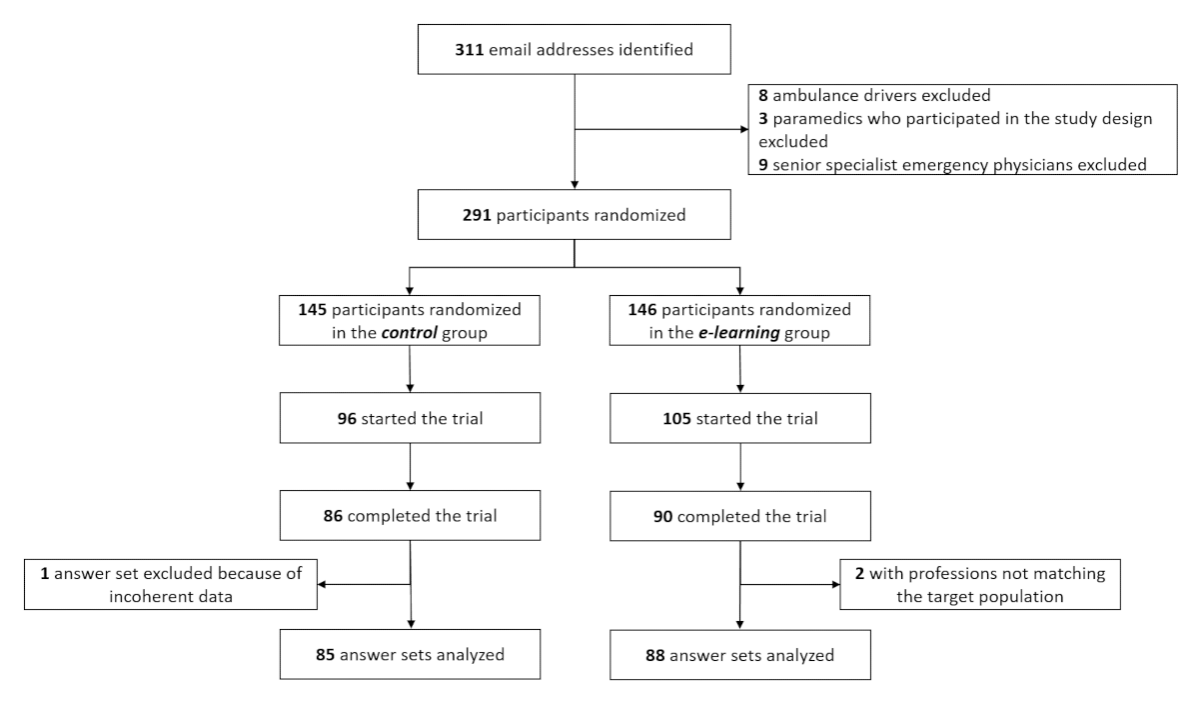
Study flowchart.

Of the 291 randomized participants, 176 (60.5%) completed the trial (Figure 2). No change was made to the web platform after the first batch of emails was sent. There was no significant downtime (the server was available more than 99% of the time throughout the study period). Just three participants were unable to complete the trial owing to technical problems, the exact nature of which could not be determined: two were not able to access the e-learning module, and one could not access the guideline (in PDF format). The study path was completed in one session by 82.7% of the participants (143/173).

The blinded data analysts (L Stuby and MA) excluded two surveys from the e-learning group as the participants’ professions did not match the target population (one “ambulance driver” and one “other”). They also excluded one survey from the control group because of incoherent answers (Multimedia Appendix 3). Participant characteristics are described in Table 1.

**Table 1:** Participant characteristics^a^.

Characteristics		Control (n=85)	E-learning (n=88)
**Profession, n (%)**			
	Student paramedic	5 (5.9)	10 (11.4)
	Emergency medical technician	11 (12.9)	12 (13.6)
	Paramedic	61 (71.8)	60 (68.2)
	Physician	8 (9.4)	6 (6.8)
Gender, female, n (%)		32 (37.6)	28 (31.8)
Age (years), median (Q1-Q3)		35 (30-42)	34 (28-40)
Professional experience (years), median (Q1-Q3)		9 (3-15)	7 (2-12)
**Prior infection prevention and control course, n (%)**			
	No/does not remember	73 (85.1)	70 (79.6)
	Yes	12 (14.1)	18 (20.4)
Coronavirus disease status, positive, n (%)		7 (8.3)	6 (6.8)
Local guideline seen, yes, n (%)		79 (92.9)	84 (95.4)
Last time guideline seen (days), median (Q1-Q3)		5 (3-10)	5 (2-8)
Specific coronavirus disease course followed, yes, n (%)		28 (32.9)	32 (36.4)

^a^Totals may not equal to 100% due to rounding.

### Main Results

There was no significant difference in baseline knowledge between groups. Though the baseline proportion of adequate PPE choice was high (75%, IQR 50%-75%), description of the donning sequence (assessed preintervention) was in most cases incorrect, as only 7 (4%) of the participants were able to reconstitute it accurately, with a similar proportion between groups. The donning sequence initially displayed in the survey was left unchanged by 7% of the participants (12/173, 6 per group).

**Figure 3:**
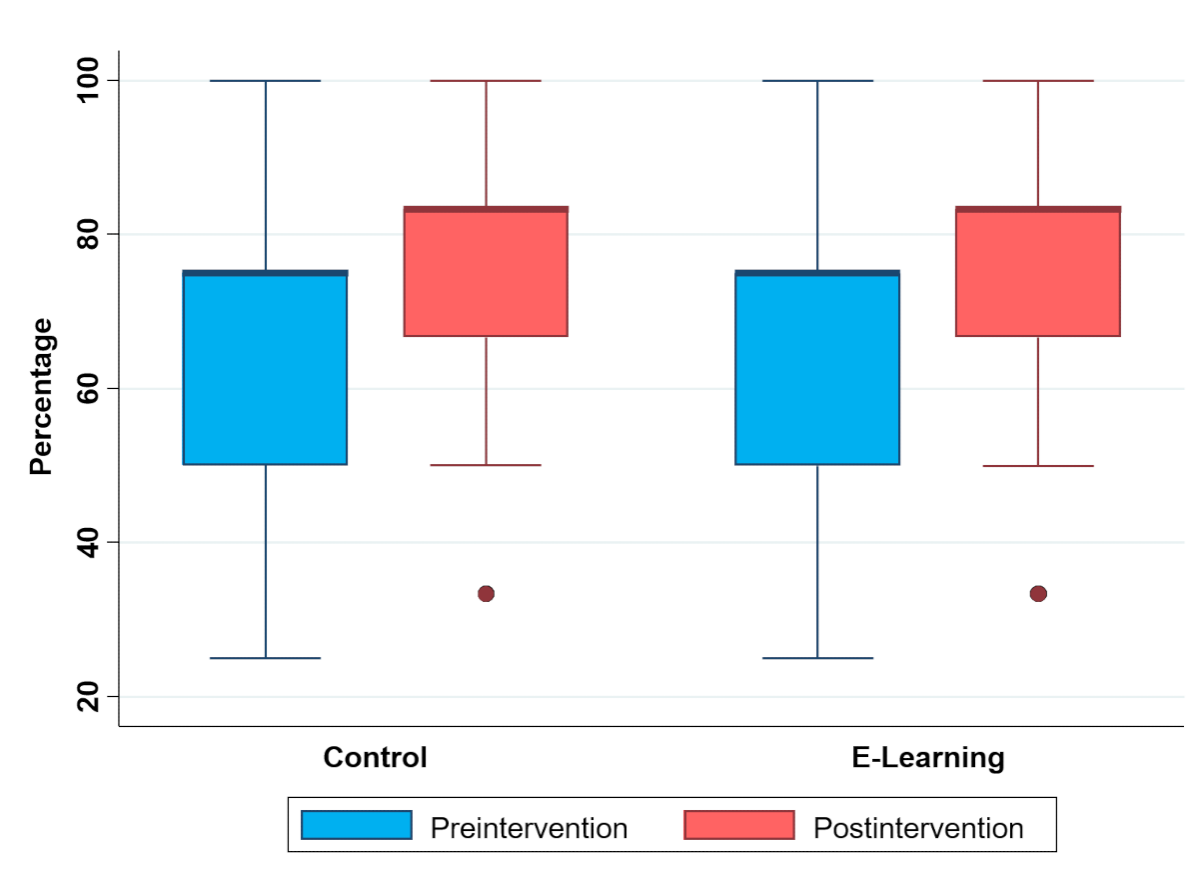
Change in proportion (%) of adequate choice of personal protective equipment.

Adequate choice of PPE was significantly increased in both groups after the intervention (*P*<.001; Figure 3). Though the median of the difference was higher in the e-learning group (17%, IQR 8%-33% versus 8%, IQR 8%-33%), it did not reach statistical significance (*P*=.27). This difference was similar regardless of stratification by profession or history of COVID-19 (Table 2). No participant was able to describe the correct doffing sequence, which was assessed postintervention. The doffing sequence originally displayed in the survey was left unchanged by 8% of the participants (14/173, 7 per group).

**Table 2:** Adequate choice of personal protective equipment (main outcome).

Outcomes		Control (n=85)	E-learning (n=88)	*P* value
Main outcome: difference in percentage of correct answers, median (Q1-Q3)		8 (8 to 33)	17 (8 to 33)	.27
**Main outcome by profession (%), median (Q1-Q3)**				
	Paramedic	8 (0 to 25)	17 (8 to 33)	.15
	Paramedic student	25 (8 to 33)	29 (25 to 33)	.49
	Emergency medical technician	25 (8 to 33)	8 (–8 to 33)	.88
	Physician	25 (13 to 38)	13 (–8 to 42)	.30
**Main outcome by coronavirus disease status (%), median (Q1-Q3)**				
	Negative for coronavirus disease	8 (8 to 33)	25 (8 to 33)	.20
	Positive for coronavirus disease	8 (–8 to 33)	0 (–8 to 17)	.37

Confidence in the ability of using PPE was identical before and after the course in the e-learning group (*P*=.27), but was significantly lower after the course in the control group (*P*=.04).

**Figure 4:**
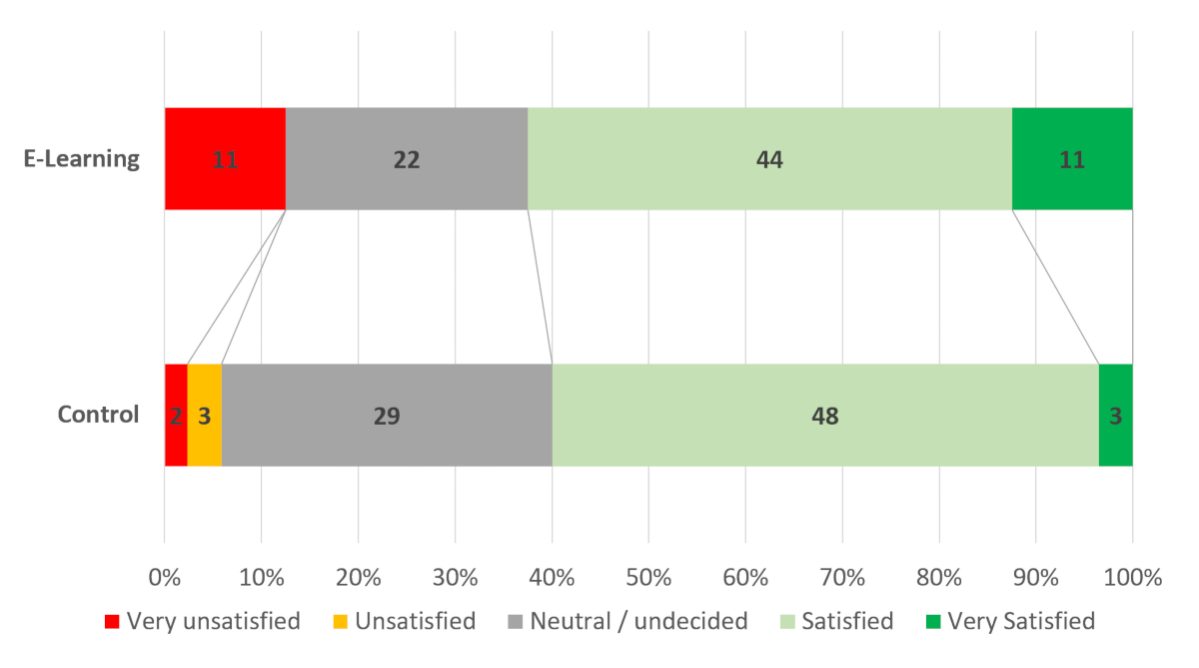
Participant satisfaction.

While most participants found the course useful (68.5%, 95% CI 61.5%-75.3%), the proportion of participants finding the course “very useful” was significantly higher (*P*=.01) in the e-learning group (33.0%, 95% CI 23.2%-42.8% versus 11.8%, 95% CI 4.9%-18.7%). Participants were generally satisfied regarding the course (60.0%, 95% CI 49.6%-70.4% for the e-learning group versus 62.5%, 95% CI 52.4%-72.6%; *P*=.28; Figure 4).

The proportion of positive comments was similar between groups (14%, 95% CI 7%-21% in the e-learning group versus 19%, 95% CI 11%-27%; *P*=.16). Participants who expressed disagreements with IPC recommendations were also evenly divided (5.7%, 95% CI 3.2%-8.2% in the e-learning group versus 4.7%, 95% CI 0.2%-9.2%; *P*>0.99).

Two post hoc sensitivity analyses were performed. The first was achieved by excluding all participants who answered they were either “very unsatisfied” or “unsatisfied” with the course. The second was performed by excluding participants whose compliance regarding COVID-19–specific IPC guidelines could be doubted; this included those who did not answer “systematic wearing of a double pair of gloves” to the question “Which of these measures is NOT one of the infection prevention measures?” (40 [45%] in the e-learning group versus 35 [41%]). These analyses did not demonstrate a significant change regarding the main outcome (*P*=.89 and *P*=.29, respectively).

## Discussion

### Main Results

In this study, the proportion of those making an adequate choice regarding PPE increased significantly after prehospital personnel followed either web-based study path. Though the median of the increase was twice that of the e-learning group, this difference was not statistically significant.

Failure to reach significance might be explained by many different factors. The high baseline proportion of adequate choice of PPE in both groups may have dampened any relative impact of the intervention as there was little room left for improvement. Although this result might seem counterintuitive given the high rate of participants who responded that they did not attend (or did not recall attending) an IPC course prior to this study, more than 90% declared they had consulted the local guidelines recently. Most participants were therefore aware of the local recommendations, and the anxiety generated by the COVID-19 pandemic probably acted as a catalyst regarding their interest in such guidelines [23].

Another factor that might partly explain the lack of a significant difference is the sample size, which limited the power to detect small differences. Nevertheless, as PPE guidelines vary not only between countries, but also between Swiss cantons, it might have been inadequate to draw conclusions from a pooled group of paramedics working in different cantons with different guidelines [24]. Moreover, though increasing the sample size would increase the chances of finding a significant difference, too small an increase would not be clinically relevant regardless of the *P* value obtained [25]. Lack of significance might therefore also be consecutive to lack of effect of the e-learning module regarding knowledge acquisition in this particular population.

Though necessary to assess participants’ knowledge and attitudes regarding PPE prior to the interventions, the first set of questions, along with the study title, might have acted as a primer and focused the participants’ attention on the specific contents that would be tested postintervention [26]. This effect might have further dampened the potential impact of the e-learning module.

The relatively low level of satisfaction displayed by the participants should also be taken into account. Though e-learning modules and serious games usually increase participant satisfaction when compared to more traditional methods [27,28], such an effect could not be found in this study. Disagreement with IPC guidelines might, at least in part, explain why some participants were dissatisfied [5,29,30]. Indeed, the vast majority of paramedics working in Geneva have had some prior training regarding the use of PPE in situations other than the COVID-19 pandemic. Preparations for high-risk transfers during the 2014 Ebola epidemic [31] and practical exercises in the context of simulated major incidents involving the presence of hazardous materials [32] have presented paramedics with many different PPE guidelines and protocols over the last few years. As the latter situations require more stringent donning and doffing procedures than those described in the COVID-19 context, some paramedics seem to feel that IPC recommendations do not go far enough. There is however a delicate balance between underprotection and overuse of scarcely available resources that must be maintained [33,34]. Moreover, it can hardly be expected to have all paramedics don maximal PPE for all interventions. This would not only considerably increase intervention time, but also make delicate interventions more difficult given the bulk of the PPE and lead to more difficult communications. It could also increase fatigue as such equipment has been shown to be quite uncomfortable [35].

### Secondary Outcomes

Though confidence in the ability to use PPE was maintained in the e-learning group, it significantly decreased after reviewing the guidelines in the control group. Participants who initially felt confident in their knowledge might have felt it was challenged after being asked specific questions [36,37]. The e-learning module probably helped restore their altered confidence, as interactive presentations, as well as gamification, have been shown to increase this feeling [38,39]. Nevertheless, most participants were unable, regardless of their assigned intervention, to reconstruct either the donning (assessed preintervention) or the doffing (assessed postintervention) sequences. This potential limitation has already been highlighted in the paper describing the development of the gamified e-learning module used in this study [13], though cut scenes were embedded to provide direct demonstration [40,41].

Though incorrect answers regarding the donning sequence are easily understandable as they might primarily result from a lack of knowledge, an inability to correctly rebuild the doffing sequence is questioning. No less than four different broad categories of causes could be involved: ineffectiveness of the teaching material, inadequate sequence, flawed means of assessing the participants’ knowledge, or disagreement with the procedure outlined in the guidelines and in the e-learning module. As this result was unforeseen, the method used in this study was unfortunately ill-suited to the evaluation of the underlying causes. Nevertheless, with less than 10% of participants having left the initially displayed sequences unchanged, a technical flaw can reasonably be ruled out.

### Limitations

Apart from the abovementioned limitations, the ever-increasing knowledge regarding SARS-CoV-2 and COVID-19 might render both the guideline and the gamified e-learning module used in this study obsolete. However, current technological tools might mitigate this effect as they allow for a quick adaptation, even of highly interactive content [42].

Another limitation is the relatively small number of questions asked pre- and postintervention. Keeping the total number of questions and the time required to complete either study path relatively low was necessary to limit attrition [16,43]. This strategy was altogether successful as dropouts amounted to less than 15% in either group, a rather lower proportion than generally reported [44]. Attrition was further limited thanks to the use of unique identifiers, which allowed as many as 30 participants (17.3%) to complete their assigned study path in more than one session.

Despite its limitations, this study also has some strengths, among which the quadruple-blind design and the relatively high response rate should be acknowledged. Moreover, as all answers were electronically recorded, there was no risk of an outcome assessment bias. Finally, as neither the control nor the e-learning path requires the physical presence of either participants or instructors, the framework used in this study could serve as the building ground for courses in the context of an epidemic or a pandemic such as the current COVID-19 situation.

### Perspectives

The larger impact such a web-based study might have had, regardless of the effect of a specific intervention such as the gamified e-learning module, should be assessed, as a possible change in PPE consumption or infection rate among prehospital providers could ensue.

The potential impact of this gamified e-learning module on less experienced and less primed participants should also be evaluated to confirm (or refute) the theories outlined in this discussion. The gamified e-learning module can thus be freely downloaded from the study website in both a web (HTML5 with Flash fallback) and SCORM (Shareable Content Object Reference Model) format.

### Conclusions

Among prehospital personnel with an already relatively high knowledge and experience regarding PPE use, both web-based study paths increased the rate of adequate choice of PPE. There was no major added value of the gamified e-learning module apart from preserving participants' confidence in their ability to correctly use PPE.

## Appendix

Multimedia Appendix 1 First email sent to participants belonging to the control group.

Multimedia Appendix 2 First email sent to participants belonging to the e-learning group.

Multimedia Appendix 3 Details of the answer set excluded by the blinded data analysts.

## Abbreviations

CHERRIES: Checklist for Reporting Results of Internet E-Surveys
CONSORT-EHEALTH: Consolidated Standards of Reporting Trials of Electronic and Mobile HEalth Applications and onLine TeleHealth
COVID-19: coronavirus disease
E-learning: electronic learning
EMT: emergency medical technician
IPC: infection prevention and control
PPE: personal protective equipment
SARS-CoV-2: severe acute respiratory syndrome coronavirus 2
SCORM: Shareable Content Object Reference Model
SMUR: Service Mobile d’Urgences et de Réanimation
