# Supplementary material for: Effect of an E-Learning Module on Personal Protective Equipment Proficiency Among Prehospital Personnel: Web-Based Randomized Controlled Trial
Source: J Med Internet Res. 2020 Aug 21;22(8):e21265. doi: 10.2196/21265 (PMC7446759; doi:10.2196/21265)
Supplement: Multimedia Appendix 1 [file jmir_v22i8e21265_app1.pdf]

Bonjour,

Comme cela vous l'a probablement déjà été annoncé, nous conduisons une étude/formation sur l'emploi des équipements de protection individuelle (EPI) dans le contexte COVID. Le contenu de cette formation étant interactif, nous vous conseillons d'employer un navigateur récent pour y accéder.

{surveyurl:4}

Durant cette formation, plusieurs questions générales comme spécifiques vous seront posées, et des vignettes cliniques vous seront présentées. Vous aurez l'occasion de lire/relire le guideline COVID le plus actuel, et, surtout, d'effectuer un module e-learning spécialement créé pour l'occasion!

Après avoir complété ce parcours, qui dure moins de 30 minutes, vous pourrez imprimer un certificat de participation (vous devrez saisir votre identité manuellement pour pouvoir générer le certificat, et elle ne pourra pas être liée aux réponses que vous avez saisies).

Nous vous remercions pour votre participation!

En cliquant sur le lien, vous acceptez de participer à l'étude. Nous vous garantissons une parfaite confidentialité.

Si Internet Explorer est votre navigateur par défaut, il vous suffit de copier le lien ci-dessus dans un navigateur tel que Chrome, Firefox ou Opera.

Pour l'équipe d'étude (n'hésitez pas à nous contacter):
- Birgit GARTNER - birgit.gartner@hcuge.ch
- Laurent SUPPAN - laurent.suppan@hcuge.ch
- Eric GOLAY - eric.golay@hcuge.ch
- Philippe COTTET - philippe.cottet@hcuge.ch
- Loric STUBY - l.stuby@gt-ambulances.ch

Pour les intéressés, la précédente étude, similaire, que nous avions menée dans le cadre de l'emploi potentiel de l'échelle NIHSS pour évaluer les AVC préhospitaliers, est disponible en pre-print (attente d'approbation) sur le site du Journal of Medical Internet Research: https://doi.org/10.2196/preprints.18358
