# Supplementary material for: Effect of an E-Learning Module on Personal Protective Equipment Proficiency Among Prehospital Personnel: Web-Based Randomized Controlled Trial
Source: J Med Internet Res. 2020 Aug 21;22(8):e21265. doi: 10.2196/21265 (PMC7446759; doi:10.2196/21265)
Supplement: Multimedia Appendix 3 [file jmir_v22i8e21265_app3.pdf]

Equipement de Protection Individuel (EPI) dans le contexte COVID

Vous êtes:

- [x] Une femme (Your answer)
- ○ Un homme

Quel est votre âge?

Veuillez ne saisir que deux chiffres.

Your answer:

10

Quelle est votre profession?

- [x] Ambulancier.e ES (Your answer)
- ○ Chauffeur.e BLS-AED
- ○ Etudiant.e Ambulancier.e
- ○ Techicien.ne Ambulancier.e (TA)
- ○ Médecin
- ○ Autre

Depuis combien d'années exercez-vous votre profession?

Veuillez ne saisir que des chiffres

Your answer:

1

▸ Pour quel service genevois travaillez-vous principalement?

- ☑ BSC - SMUR/REGA (Your answer)
- ○ ACE
- ○ CERN
- ○ GTA
- ○ SAR
- ○ SAG
- ○ SK
- ○ SIS
- ○ SSLIA

▸ Avez-vous déjà suivi un cours sur la prévention et le contrôle de l'infection (PCI)?

- ☑ Non (Your answer)
- ○ Oui
- ○ Je ne me souviens pas

▸ Quel est votre statut COVID?

- ☑ Je ne souhaite pas répondre à cette question (Your answer)
- ○ COVID négatif / pas testé
- ○ COVID positif - en isolement actuellement
- ○ COVID positif - guéri et de retour au travail

▸ Avez-vous déjà vu le guideline COVID préhospitalier genevois?

- ☑ Non (Your answer)
- ○ Oui
- ○ Je ne me souviens pas l'avoir vu

▸ A quand remonte votre dernière consultation du guideline COVID (en jours)?

Une valeur approximative suffit.

Your answer:

30

▸ Avez-vous eu une formation spécifique relative au COVID dans votre service préhospitalier?

- ☑ Oui (Your answer)
- ○ Non

▸ Sous quelle forme cette formation vous a-t-elle été proposée?

- ☑ Atelier pratique (Your answer)
- ☐ Information par courrier électronique
- ☐ Webinar
- ☐ E-Learning
- ☐ Autre formation en ligne
- ☐ Annonce orale
- ☐ Formation présentielle
- ☐ Visioconférence
- ☐ Vidéo
- ☐ Autre

▸ Concernant la possibilité de contracter/transmettre l'infection, à quel point êtes vous inquiet:

	Pas inquiet	Peu inquiet	Inquiet	Très inquiet
Pour vous-même	☐	☐	☐	☑
Pour vos proches	☐	☐	☐	☑
Pour vos patients	☐	☐	☐	☑

▸ Vous sentez-vous confiant dans vos connaissances d'utilisation des équipements de protection indivduels?

- ☑ Très confiant (Your answer)
- ○ Pas du tout confiant
- ○ Peu confiant
- ○ Neutre / indéterminé
- ○ Assez confiant

▸ Les termes SARS-CoV-2 et COVID-19 sont interchangeables

- ☑ Faux (Your answer)
- ○ Vrai

▸ Une infection à SARS-CoV-2 est toujours symptomatique

- ☑ Faux (Your answer)
- ○ Vrai

▸ Seuls les patients âgés, ou souffrant de comorbidités importantes, vont présenter des complications graves pouvant aller jusqu'au décès

- ☑ Faux (Your answer)
- ○ Vrai

▸ La transmission peut se faire par (plusieurs réponses possibles):

- ☑ Contact direct avec le patient (Your answer)
- ☑ Contact avec des surfaces touchées par le patient (Your answer)
- ☑ Goutelettes (éternuements, postillons) (Your answer)
- ☑ Par aérosolisation / nébulisation (Your answer)

▸ Après avoir été infecté, les symptômes se déclarent généralement (chez la majorité des patients) après:

- ☑ 5-6 jours (Your answer)
- ○ Moins de 24 heures
- ○ 1-2 jours
- ○ 3-4 jours
- ○ 7-8 jours
- ○ 9-10 jours
- ○ 11 jours ou plus

▸ Vignette Clinique

Il est 5 heures du matin. Vous êtes engagés par la CASU-144 pour un homme de 81 ans, connu pour hypertension artérielle, présentant une dyspnée brutale peu avant l'appel. Aucune autre information n'est disponible initialement. Le SMUR est indisponible. Le patient est à son domicile, au 2ème étage d'un immeuble en ville.

▸ 1. En plus de l'équipement de protection standard, comment allez-vous vous équiper avant d'accéder au patient si vous êtes leader (plusieurs réponses possibles)?

- ☑ Masque FFP-2 (Your answer)
- ☑ Tenue Tyvek avec capuche (Your answer)
- ☑ Lunettes de protection (Your answer)
- ☐ Masque chirurgical
- ☐ Tenue Tyvek sans capuche

▸ 2. En plus de l'équipement de protection standard, comment allez-vous vous équiper si vous êtes second et que vous restez à distance (plusieurs réponses possibles)?

- ☑ Masque chirurgical (Your answer)
- ☑ Tenue Tyvek sans capuche (Your answer)
- ☐ Masque FFP-2
- ☐ Tenue Tyvek avec capuche
- ☐ Lunettes de protection

▸ Vignette clinique

Vous intervenez en P1 pour état confusionnel aigu au domicile d'une femme de 80 ans, qui présente de la fièvre avec une toux sèche en aggravation depuis 6 jours. Depuis le pas de la porte vous constatez une FR>25/min.

▸ Quel équipement de protection spécifique devez-vous mettre en tant que leader?

- ☑ Masque FFP2, combinaison avec capuchon, lunettes, gants (Your answer)
- ○ Masque chirurgical, combinaison sans capuchon, gants
- ○ Masque chirurgical, gants
- ○ Masque FFP2, combinaison sans capuchon, lunettes, gants
- ○ Masque FFP2, surblouse, lunettes, gants

▸ Quel EPP spécifique devez-vous mettre en tant que second si vous restez à distance?

- ☑ Masque chirurgical, combinaison sans capuchon, gants (Your answer)
- ○ Masque chirurgical
- ○ Masque chirurgical, combinaison avec capuchon, lunettes, gants
- ○ Masque FFP2, combinaison sans capuchon, lunettes, gants
- ○ Pas d'EPP spécifique si on respecte la distance > 3m

▸ Quelle est la séquence à suivre si vous devez revêtir un équipement de protection individuel pour prendre soin d'un patient suspect de COVID en insuffisance respiratoire?

1. Préparation d'une poubelle pour recueillir l'équipement en fin de déshabillage
2. Désinfection des mains et mise d'une paire de gants non stériles
3. Mise en place des lunettes de protection
4. Mise en place du masque FFP-2 et test d'étanchéité
5. Equipement avec une tenue Tyvek, capuche comprise

▸ Guideline préhospitalier COVID-19

Avant de continuer, merci de consulter le guideline préhospitalier le plus récent en cliquant sur le lien ci-dessous:

Guideline préhospitalier COVID version 1.11c (https://smur.hug-ge.ch/sites/smur/files/guideline_covid19_1_11c.pdf)

Après avoir consulté le guideline, fermez l'onglet le contenant pour revenir à cette page et continuer le parcours.

▸ Avez-vous accédé au guideline préhospitalier COVID?

- [x] Oui, je confirme avoir eu accès au guideline. (Your answer)
- ○ Non, je n'ai pas pu avoir accès au guideline

▸ Module E-Learning

Cliquez ici pour accéder au module e-learning (https://www.cardiomobile.ch/coronavirus/story.html)

ATTENTION: pour les utilisateurs des **HUG**, cliquez sur ce lien (compatible internet explorer) (https://www.cardiomobile.ch/coronavirus/flash/story.html)

Après avoir consulté le module, vous pouvez simplement fermer l'onglet le contenant pour revenir à la formation.

▸ Avez-vous accédé au module e-learning?

- [x] Oui, je confirme avoir eu accès au module e-learning (Your answer)
- ○ Non, je n'ai pas pu avoir accès au module e-learning

▸ Vignette

Il est 18 heures. Vous êtes engagés par la CASU-144 pour une détresse respiratoire avec suspicion de COVID-19 chez un patient de 27 ans, vivant à domicile avec sa compagne. Le patient et sa compagne ont tous deux été dépistés une semaine auparavant, car ils présentaient une toux sèche et un état fébrile. Le frottis du patient est revenu négatif, tandis que celui de sa compagne est revenu positif pour un SARS-CoV-2.

▸ Ce patient peut-il tout de même être atteint du COVID-19?

- ☑ Oui (Your answer)
- ○ Non

▸ 1. En plus de l'équipement de protection standard, comment allez-vous vous équiper avant d'accéder au patient si vous êtes leader?

- ☑ Masque FFP-2 (Your answer)
- ☑ Tenue Tyvek sans capuche (Your answer)
- ☑ Lunettes de protection (Your answer)
- ☐ Masque chirurgical
- ☐ Tenue Tyvek avec capuche

▸ 2. En plus de l'équipement de protection standard, comment allez-vous vous équiper si vous êtes second et que vous restez à distance?

- ☑ Masque chirurgical (Your answer)
- ☐ Masque FFP-2
- ☐ Tenue Tyvek sans capuche
- ☐ Tenue Tyvek avec capuche
- ☐ Lunettes de protection

▸ Quelle séquence de déshabillage devez-vous suivre une fois la prise en charge de votre patient suspect de COVID terminée?

1 Mise du matériel médical en contact avec le patient (stéthoscope, etc.) dans un sac prévu à cet effet

2 Enlever les gants et se désinfecter les mains

3 Ouvrir la tenue Tyvek et se désinfecter les mains

4 Retirer le capuchon sans toucher les parties externes, puis retrousser progressivement la Tyvek jusqu'aux chaussures

5 Retirer les chaussures, puis la Tyvek

6 Se désinfecter les mains et disposer des lunettes de protection

▸ Parmi ces mesures, quelle est celle qui NE FAIT PAS partie des mesures de prévention de l'infection (une seule réponse possible)?

- ☑ Le port systématique d'une double couche de gants (Your answer)
- ○ Une technique de déshabillage maitrisée en enroulant la combinaison vers l'intérieur
- ○ L'hygiène des mains à l'aide d'une solution hydro-alcoolique
- ○ La désinfection des lunettes de protection à l'aide d'une compresse imbibée d'éthanol à 70%

▸ Vignette clinique

Il est 15 heures. Vous êtes engagés sur un transport secondaire d'un patient en sepsis sévère depuis les urgences des HUG pour l'hôpital de La Tour (frottis négatif). Le patient, un homme de 43 ans, a été admis pour des frissons présents depuis le matin même, précédés par une douleur lombaire gauche depuis 48 heures. Il est toujours hautement fébrile (température centrale: 40.2°C) et a été mis sous antibiotiques par voie intra-veineuse. Il ne tousse pas, et ne pense pas avoir été en contact avec des personnes victimes du COVID.

▸ 1. En plus de l'équipement de protection standard, comment allez-vous vous équiper avant d'accéder au patient si vous êtes leader?

- ☑ Masque chirurgical (Your answer)
- ☑ Tenue Tyvek sans capuche (Your answer)
- ☐ Masque FFP-2
- ☐ Tenue Tyvek avec capuche
- ☐ Lunettes de protection

▸ 2. En plus de l'équipement de protection standard, comment allez-vous vous équiper si vous êtes second et que vous restez à distance?

- ☑ Masque chirurgical (Your answer)
- ☐ Masque FFP-2
- ☐ Tenue Tyvek sans capuche
- ☐ Tenue Tyvek avec capuche
- ☐ Lunettes de protection

▸ Vignette clinique

Vous intervenez avec le SMUR au domicile d'une femme de 40 ans connue pour HTA, qui aurait présenté une dyspnée soudaine suivie d'une syncope. A votre arrivée sur site massage cardiaque en cours par un voisin de palier.

▸ Quel équipement de protection le leader doit-il porter?

- [x] Masque FFP2, combinaison avec capuchon, lunettes, gants (Your answer)
- ○ Masque chirurgical, combinaison sans capuchon, lunettes gants
- ○ Masque FFP2, surblouse, lunettes, gants
- ○ Masque FFP2, lunettes, gants
- ○ Masque chirurgical, gants

▸ Quel équipement de protection le médecin SMUR doit-il porter?

- [x] Masque FFP2, combinaison avec capuchon, lunettes, gants (Your answer)
- ○ Masque chirurgical, combinaison sans capuchon, lunettes gants
- ○ Masque FFP2, surblouse, lunettes, gants
- ○ Masque FFP2, lunettes, gants
- ○ Masque chirurgical, gants

▸ Pour éviter de contracter le COVID, la phase la plus critique est:

- [x] Ces phases sont aussi critiques l'une que l'autre (Your answer)
- ○ L'habillage
- ○ Le déshabillage

▸ Vous sentez-vous confiant dans vos connaissances d'utilisation des équipements de protection individuels?

- ☑ Très confiant (Your answer)
- ○ Pas du tout confiant
- ○ Peu confiant
- ○ Neutre / indéterminé
- ○ Assez confiant

▸ Pensez-vous que cette formation vous a été utile / vous sera utile?

- ☑ Oui, très utile (Your answer)
- ○ Non, inutile
- ○ Non, peu utile
- ○ Ne sais pas / indéterminé
- ○ Oui, utile

▸ A quel point êtes-vous satisfait·e de ce parcours de formation?

- ☑ Très satisfait (Your answer)
- ○ Très insatisfait
- ○ Insatisfait
- ○ Neutre / indécis
- ○ Satisfait

▸ Avez-vous des remarques/commentaires à nous communiquer?

No answer given.
